# Water-Based Indium Tin Oxide Nanoparticle Ink for Printed Toluene Vapours Sensor Operating at Room Temperature

**DOI:** 10.3390/s18103246

**Published:** 2018-09-27

**Authors:** Jan Maslik, Ivo Kuritka, Pavel Urbanek, Petr Krcmar, Pavol Suly, Milan Masar, Michal Machovsky

**Affiliations:** Centre of Polymer Systems, University Institute, Tomas Bata University in Zlin, trida Tomase Bati 5678, 760 01 Zlin, Czech Republic; maslik@utb.cz (J.M.); urbanek@utb.cz (P.U.); pkrcmar@utb.cz (P.K.); suly@utb.cz (P.S.); masar@utb.cz (M.M.); machovsky@utb.cz (M.M.)

**Keywords:** Indium tin oxide, nanoparticle, inkjet ink, material printing, dimensionless number, gas sensor, room temperature

## Abstract

This study is focused on the development of water-based ITO nanoparticle dispersions and ink-jet fabrication methodology of an indium tin oxide (ITO) sensor for room temperature operations. Dimensionless correlations of material-tool-process variables were used to map the printing process and several interpretational frameworks were re-examined. A reduction of the problem to the Newtonian fluid approach was applied for the sake of simplicity. The ink properties as well as the properties of the deposited layers were tested for various nanoparticles loading. High-quality films were prepared and annealed at different temperatures. The best performing material composition, process parameters and post-print treatment conditions were used for preparing the testing sensor devices. Printed specimens were exposed to toluene vapours at room temperature. Good sensitivity, fast responses and recoveries were observed in ambient air although the n-type response mechanism to toluene is influenced by moisture in air and baseline drift was observed. Sensing response inversion was observed in an oxygen and moisture-free N_2_ atmosphere which is explained by the charge-transfer mechanism between the adsorbent and adsorbate molecules. The sensitivity of the device was slightly better and the response was stable showing no drifts in the protective atmosphere.

## 1. Introduction

A great deal of effort has been spent on describing the mechanism responsible for the gas sensing of metal oxide nanomaterials as active components of sensing devices [1,2,3]. In order to boost the sensitivity and achieve better selectivity and stability of these devices, sophisticated hierarchical and hybrid nanostructured materials have recently been prepared and tested, e.g., [4,5]. On the other hand, research papers focused on the fabrication and improvement of sensing devices are less frequent [6,7].

Nevertheless, several well established general methods are known for preparing thin and thick metal oxide nanomaterial films which can be used in the fabrication of gas monitoring devices. The most commonly used techniques can be divided into two main groups. The first is a plethora of gas phase fabrication techniques (PVD, CVD or thermal vapour transport methods). The second group of methods relies on liquid phase employing fabrication techniques such as sol-gel, spray pyrolysis, dip-coating, spin-coating and material printing methods [8,9,10].

The above-mentioned conventional deposition techniques, gas phase and in-situ liquid phase growing mechanism techniques are strictly constrained by various physical and chemical parameters dictating the utilization of special equipment and the exposition of the substrate to reaction environment. The disadvantages of these techniques include, in most cases, the need to using vacuum technology, stencils and masks or subsequent separating, removing or etching processes. Such problems can be easily overcome by using material printing technology if the material can be prepared in form of a printable ink, paste, and powder or others in suitable formulation. Material printing technologies (including screen-printing, roll-to-roll printing, gravure printing and ink-jet printing) are widely used especially in electronics (fabrication of conductive circuits and paths, solar cells, light-emitting and sensing devices, antennas, membranes etc.). Their applications result in simplified and accelerated fabrication processes. These low-cost technologies are based on depositing functional materials onto a used substrate such as glass or transparent and flexible foils, textile materials, ceramics or metal wafers. Unlike other printing methods, ink-jet printing does not require any master form, stencils or masks; therefore, it allows instantaneous and rapid designs and prototyping with no delay between digital motive generation and material deposition. The ink-jet printing is an interesting and versatile method to make controlled and localized deposition of functional materials with suitable geometry on various substrates at low processing temperatures [11,12,13,14]. The printed patterns are designed by common computer programs and can be saved as simple bitmap images for printing. It is possible to use a wide range of inorganic and organic materials, including inks based on metal or metal oxide nanoparticles and polymer solutions [15,16,17]. The size of the oxide nanoparticles, their dispersion in a proper liquid medium and a suitable dispersion stabilization, viscosity and the surface tension of the ink composition for jetting are vitally important and challenging parameters to be developed for the required flow of the ink through the nozzles of a printing head yielding the proper generation and ejection of droplets, which is the limiting factor of this material deposition printing process.

Transparent conductive oxides (among them indium tin oxide—ITO being the most widely used) are indispensable materials in fabrication of optoelectronic devices and can be used as a semi-conductive material for gas detecting sensors based on the resistance variation due to its exposure to the target gas. Films made from ITO (In_2_O_3_:10 wt% SnO_2_) have been extensively studied in recent years because they exhibit a relatively rare combination of high visible transmission and significant electrical conductivity, high substrate adherence, good hardness, and chemical inertness [18]. The applicability of this degenerate n-type semiconductor as a thin film sensory device deposited by various techniques (such as the thermal evaporation technique, sputtering, screen-printing) has been widely reported with respect to sensing reducing and oxidizing gases and organic compounds (NH_3_, CO, H_2_, NO_2_, toluene etc.) [6,7,19,20,21]. For example, toluene is a widely used organic solvent and one of the significant contributors to indoor air pollution with serious safety concerns. A high temperature operating (at 230 °C) ITO thin film device for toluene vapours sensing was prepared by sputter coating recently [19].

Some applications of particulate ITO materials have been reported [15,22], although only rarely for sensing devices. It has already been demonstrated that the screen printing of organometallic precursor paste followed by firing at high temperatures (600 °C) for a relatively long time (for 40 min at optimum for ITO crystallization) can be utilized for sensor fabrication [6]. The latest efforts demonstrated the suitability of screen printing for pastes directly composed from ITO nanocrystals and binders for sensor fabrication [23,24].

To the best of our knowledge, there is no report available in literature concerning the preparation of an ITO based sensor directly prepared from ITO nanopowder with the aid of an ink-jet material printing. We are convinced, that it is possible to use this method earning all benefits it can offer and the printing process is in focus of our study. Moreover, a second challenge is preparing a device operating at room temperature, as this may be of prime importance for applications in bio-related fields, wearable electronics, energy saving approaches and heating minimization in already existing technologies, thermal camouflage etc. In this work, we describe the development of an ITO nanoparticle based waterborne dispersion for the deposition of films by ink-jet printing technology and a demonstration of the low temperature response of the prepared sensors upon exposure to saturated toluene vapours. The sensor was tested in ambient laboratory conditions as well as under a pure nitrogen atmosphere.

## 2. Materials and Methods

### 2.1. Formulation and Characterization of Indium Tin Oxide Inks

ITO nanoparticle based aqueous inks were prepared in the form of dispersions. The proper amount (weighed) of indium tin oxide nanopowder <50 nm particle size (Sigma-Aldrich spol. s r.o., Prague, Czech Republic) was mixed with the optimum ratio of polymeric dispersing agent and silicon surfactant provided by BYK-Chemie, ALTANA (Disperbyk^®^-190 and Byk^®^-348). The concentration of both these additives was set to be slightly above their CMCs. Ethylene glycol was chosen for modifying the density and viscosity of the dispersion (Sigma-Aldrich product). Ethylene glycol (EG) also provides a high boiling and humectant component of the solvent system. The dispersions were mixed for several hours in a sealed flask and were sonicated for 30 min by UZ Sonopuls HD 2070 homogenizer and filtered through a 0.22 μm PTFE filter to remove aggregates and agglomerated particles before filling the cartridge (n.b.). The concentrations of the ITO nanoparticles in the prepared inks were set to 10, 15, 20 and 25 wt% to compare the overall performance of each.

The viscosity of the inks was determined at the low-shear rate by rolling ball Microviscometer Lovis 2000 ME (1.59 mm capilary) and the density was measured by Density meter DMA 5000 M (Anton Paar s.r.o., Prague, Czech Republic). The surface tension of the ITO ink was measured by Tensionmeter K100MK3 (Krüss GmbH, Hamburg, Germany) based on the Wilhelmy plate method.

The thermogravimetric analysis was performed using a TA Q500 thermal analyzer working in TGA mode with a programmed constant temperature growth rate of 10 °C per minute and a nitrogen atmosphere with a total flow of 100 sccm.

### 2.2. Sensor Fabrication and Printing Conditions

The patterns were designed in a basic Windows program in a scale corresponding to 15 mm × 15 mm, then saved as a bitmap image and loaded in Drop Manager Software. The sensor films were fabricated by a material piezoelectric inkjet printer Fujifilm Dimatix DMP 2831 Series (nozzle diameter 21.5 µm and 10 pL nominal drop volume cartridge, printer and consumables provided by FUJIFILM Dimatix Inc., Lebanon, NH, USA) on microscope glass slides, which were cleaned in de-ionized water, acetone and isopropanol and dried by flowing air and in a vacuum oven. The optimum printing conditions were found and used as follows: the driving voltage at the nozzles during the printing process was in the range of 25–27 V with a jetting frequency of 1 kHz, a nozzle temperature of 32 °C and a plate temperature of 50 °C, the spacing of droplets was defined to 30 μm which is equivalent to a resolution of 847 dpi. The films were made by one printing run (one layer).

**Figure 1:**
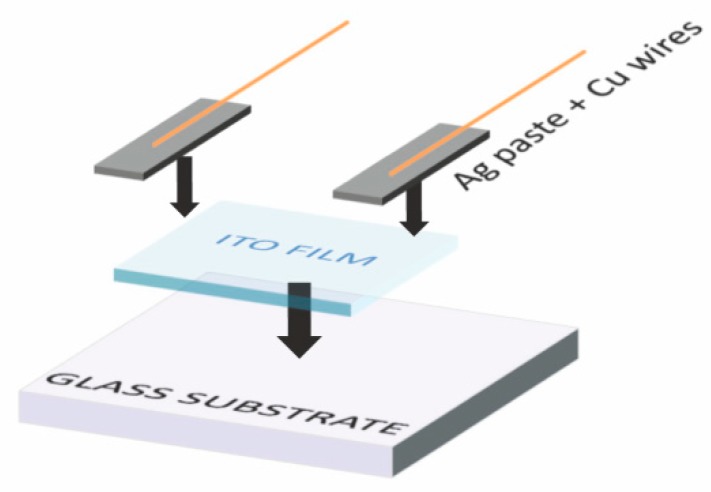
Schematic design of the sensing device.

The printed films were dried in an oven at 60 °C for 30 min and then annealed in a muffle furnace. The annealing temperatures were chosen to clarify the effect of annealing to 400, 500 and 600 °C in an ambient air atmosphere. The sample was always inserted into the cool oven and the temperature program was set to maximum rate increase. The desired temperature was achieved within a few minutes and then the temperature was kept constant for 30 min. The oven was then left to naturally cool down to the laboratory temperature before the samples were taken out. The electrodes were connected to the copper wires by the highly conductive silver paste COATES XZ250 which was dried at 120 °C for 20 min. The design of the sensing device is schematically depicted in Figure 1.

### 2.3. Films Characterization

The general quality, contiguity and compactness of the printed films were preliminary observed by the microscope LEICA DVM2500 Digital Camera. The thickness of the printed films was measured in contact mode by the stylus profiler Dektak XT (Bruker, Billerica, MA, USA) provided by a diamond stylus (25 μm radius). The electrical resistivity of the printed patterns was measured using a four-point probe (Van der Pauw method). The surface morphology was examined by the scanning electron microscope Nova NanoSEM 450 in vacuum and by the atomic force microscope Dimension ICON (Bruker) under an ambient condition at three different areas of 25 μm^2^ in PeakForce mode by the ScanAsyst–Air probe whose tip has a nominal radius of 2 nm. The surface roughness was calculated and averaged from obtained data using NanoScope Analysis 9.1. The current mapping was investigated through the PeakForce tunneling module working in semi-contact mode with the PFTUNA probe whose tip has a nominal radius of 25 nm coated by conductive platinum/iridium and was used to observe the current signal distribution on the surfaces of 25 μm^2^ of annealed thin films. The applied DC bias between the electrically conductive tip and the sample was set to 100 mV. Both a scan rate of 0.5 Hz and a resolution of 512 lines were used in all cases.

### 2.4. Sensor Performance

The response of the sensors was measured using the multimeter UNI-T HC-UT71D with interface software that records observed resistance. The changes between the “On” and “Off” sensor states upon exposure to the vapours of toluene and the removal from the vapours were measured. The experiments were carried out at an ambient atmosphere in the laboratory as well as in a nitrogen atmosphere (less than 1 ppm of O_2_ and H_2_O) in the glove box GP Campus (Jacomex).

Wherever indicated in this article, the room or laboratory temperature means (24 ± 2) °C unless stated more precisely. The laboratory is air conditioned.

## 3. Results and Discussion

### 3.1. Ink Composition Development

The most challenging step throughout the fabrication process of such a device as described in the introductory part is the formulation of the ink. It is necessary to consider that the parameters of the final printed layer have to meet all necessary chemical and physicochemical criteria to assure the compatibility of the ink with the substrate, the jetting performance and the storage stability of the suspension while preparing the ink composition. It is a multidimensional parameter space that must be researched and in which an optimum between all (and sometimes contradictory) requirements imposed on the material and its processing with the given equipment must be found. The crucial parameters enabling optimal printability are therefore ink density, viscosity, surface tension, the stability of the suspension and the size of the nanoparticles, which is partially related to the final resistance and the response of the sensing layer [16,25]. The key properties determining good processability by the used ink-jet printer Dimatix DMP-2800 Series are listed by the producer for disposable cartridges with printing heads as follows. The ink fluid viscosity shall be kept in a range of 10–12 mPa·s and its surface tension in a range of 28–42 mN·m^−1^. Furthermore, it is suggested to use a drop velocity in a range of 7–9 m∙s^−1^ as the first guess [26], however, according to literature and experience in this study, a fluid ejection velocity of about 6 m∙s^−1^ is commonly used. It is also recommended to filter all fluids to 0.2 μm, because particles bigger than 1/100 of the nozzle diameter may cause nozzle clogging. The first two parameters may be substantially changed by the ink composition and the size of the nanoparticles must be chosen to be smaller than the critical level. The processing parameters, namely the fluid ejection velocity and the droplet formation, are controlled by using waveform; however, they can be only slightly varied in comparison with the relatively free choice of the ink viscosity and the surface energy. According to the producer, the viscosity range may be extended from 1 mPa·s for water-like fluids up to 30 mPa·s which is declared as the highest viable for the printer. Similarly, a surface tension of about 70 mN·m^−1^ represents the upper limit for printing with a given machine while the lower limit is 20 mN·m^−1^. The original ink composition was experimentally developed within this space of parameters by the trial-error method of changing and alternating always only one variable until a satisfactory performance of the process was achieved. The properties of developed inks with variable nanoparticle loading are listed in Table 1. The surface tension of the used ink compositions is compatible with the surface characteristics of the chosen substrate. The viscosity and the surface tension under given dynamic conditions did not result in the optimum formation of single droplets only, but the system worked in the regime of the one satellite droplet formation that merges with the main droplet during its flight before hitting the surface. Although this is a suboptimum process, it yielded good printing quality while other parameters were relatively easily kept at sufficient levels too.

**Figure 2:**
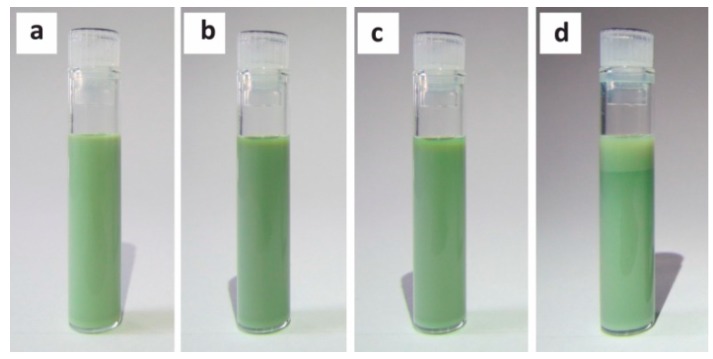
Demonstrating of the achieved stability and the sedimentation progress of the ink composition (25 wt% loading) over time (**a**) t = 0 h, (**b**) t = 24 h, (**c**) t = 72 h, (**d**) t = 10 days.

The stability of the prepared nanodispersions represents another extremely important issue for their application as inks. Sufficient shelf life is a great advantage and at least easy redisperseability must be assured for any practical utilization of inks. Due to the above-mentioned requirements the optimal amount and ratio of the two additives was determined based on the critical micelle concentration in an aqueous medium. The surface tension was controlled by using a wetting agent which produces a significant decrease in the surface tension of the aqueous system and therefore particularly improves substrate wetting and levelling. The achieved stability of the ink compositions guaranteed their safe use within the timescale of several days and is displayed in the example in Figure 2. The dissolved macromolecules of non-ionic surfactant in the dispersion medium avoid the flocculation of nanoparticles by means of steric stabilization through the functional groups having an affinity to the particle surface. The hydrophilic segments form a repulsive layer suppressing the coagulation of the nanoparticles present and stabilize them. On the role and effects of the dispersant agents in ink-jet inks, the reader is referred to Soleimani-Gorgani [27] and Young-Sang Cho [28] for further details.

The composition and processing properties of used inks with the inkjet printer DMP Dimatix 2800 Series can be analyzed rationally with the use of dimensionless criteria. Utilization of this analysis greatly enhanced the original trial-error development of the ink and accelerated optimization of the ink printing conditions. The reader is referred to the comprehensive works of Shlomo Magdassi [16], Zhidong Pan [29] and E. Kim and J. Baek [30]. According to the literature, the following criteria are the most important. Generally, the printability range used to be determined by a *Z* number, which is the inverse of the Ohnesorge number (*Oh*). The recommendations for the optimum range of the Z number vary from 1–10 up to 4–14. However, it was found that the *Z* number (or obviously *Oh* number too) alone is insufficient for describing the droplet formation dynamics because all the terms describing dynamic effects are cancelled in its formula

(1) $$Z=\frac{Re}{\sqrt{We}}=Oh^{-1}$$

and only the material constants and characteristic length remain [30]. Actually, the characteristic length (*A*) is a tool-related property since it is a printing nozzle square shaped orifice characteristic diameter, i.e., the side size of the square (*A* = 21.5 µm). Therefore, other important non-dimensional parameters such as the Reynolds number (*Re*), the Weber number (*We*), and the capillary number (*Ca*) should also be taken into consideration to complete the material-process-tool parameter-related triad. *Re* represents the ratio between the viscous and the inertial forces in moving fluid, *We* is dependent on the ratio between the inertia and the surface tension, *Oh* reflects the physical properties of the liquid (the viscous forces, the inertial and the surface tension forces) and the size scale of the nozzle, but is independent of the driving conditions, and *Ca* is the ratio of the viscous force to the capillary force. Table 2 summarises the calculated dimensionless criteria of the prepared inks with the use of ink ejection fluid velocity finally estimated with the help of analysis discussed below. The influence of gravity is considered as being of very low relevance on the investigated range of parameters (including the stand-off being 1 mm) and therefore is not considered in this analysis. The formulas and description of used variables are given in Appendix A—Used equations for dimensionless criteria.

**Figure 3:**
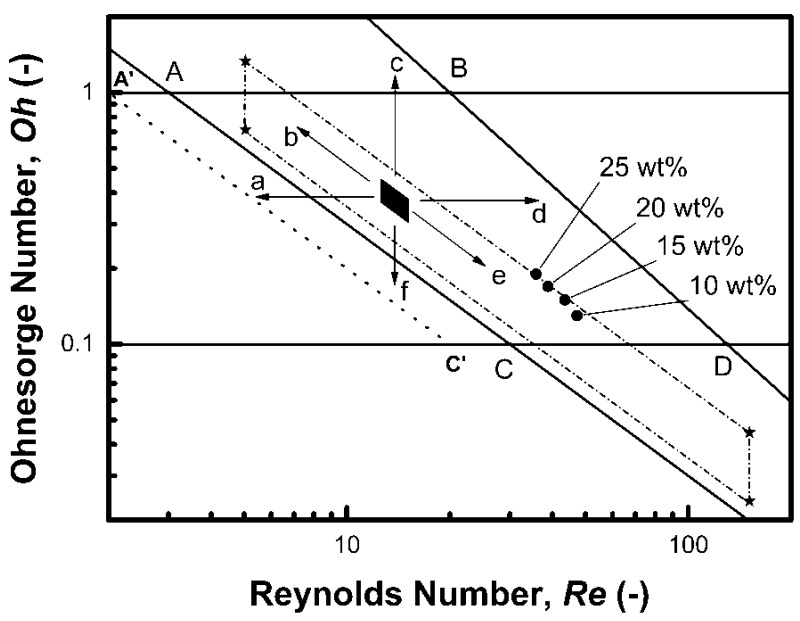
Map of *Oh* and *Re* dimensionless correlations space for a printing process with the printability area ABCD replotted according to McKinley and Renardy [31]. For a detailed description please see text.

The prepared suspensions were intentionally theoretically treated as Newtonian fluids only, neglecting the eventual weak viscoelasticity. Such an approach simplifies the problem radically; however, it can be applied until it produces predictions comparable with experiment. According to an analysis of ink-jet printing regimes by E. Kim and J. Baek [30], all prepared inks fall into the “regime II” which is characterized by relatively small Ca and large We numbers. These conditions are manifested by a satellite formation and the merging of the satellite with the main droplet during its flight, thus fulfilling the definition of good printability when “a single drop is formed either directly without a second pinch-off or the satellite drop merges with the main drop within its travel distance less than 20 times A forming thus a single drop”. Referring back to literature sources, a simpler and earlier solution involving all material-tool-process characteristics may be found in the work of McKinley and Renardy [31] who redrew the schematic diagram originally constructed by Derby [32] to show the field of parameters for stable operations of drop on demand inkjet printing by using logarithmic coordinate system defined by plotting Ohnesorge against Reynolds number. The graph constructed with the help of their definition of printability boundaries is presented in Figure 3. The quadrangle ABCD defines a region, in which the particular fluids are printable and single drop formation may be achieved or merging with the satellite can be expected. The diagram does not resolve between regime I and II as defined later by E. Kim and J. Baek [27].

The purpose of printing process optimization is an achievement of the good printability conditions which is an absolute prerequisite for obtaining good-quality thin film layer including its precise location, resolution and functionality. We found it to be useful to discuss the printing parameters according to the suggestions of the printer producer and compare them with our experimental procedure. The small black full quadrangle in the center of the ABCD area in Figure 3 represents the optimum printability space for the Dimatix printer (i.e., viscosity 10–12 mPa·s and the surface tension in the range of 28–42 mN·m^−1^) while the typical value of the density of our suspensions varies slightly between 1.1 g∙mL^−1^ and 1.3 g∙mL^−1^, therefore 1.2 g∙mL^−1^ was chosen for the model. The droplet velocity can be varied by the waveform process control; nonetheless our typical final optimum value 6 m∙s^−1^ is taken into account in this analysis. Finally, the characteristic length *A* is strictly given by the nozzle geometry in the used printing heads and cannot be changed at all. The changes of the variables involved in the dimensionless criteria result in typical shifts or extensions of this parameter space in the directions indicated by the six arrows marked from **a** to **f**. Decreasing the fluid velocity corresponds to the direction **a** while the use of higher velocity shifts the area in the direction **d**. Increasing the surface tension shifts the parameter space border downwards in direction **f** while decreasing the surface tension results in the shift upwards in direction **c**. Increasing the fluid viscosity results in the shift along the arrow **b** and decreasing the viscosity extends the area in the direction of the arrow **e**. Changes in the characteristic length or the density will result in a diagonal shift too, however, the slope will be −1/2. The full extension of the printability span according to the extreme viscosities and the surface tensions declared by the printer producer is indicated by the quadrangle with asterisks in its corners and the short dash dot sides. The position of the prepared inks is marked by the four full circle data points labelled by ITO concentrations. It must be noted that the points are aligned along a virtual line, which has slope −1 and represents a line with a constant value of *We*^1/2^ of about 6.6 which corresponds to the average *We* number value from Table 2 of about 43.5. Although a good printability was finally achieved for all compositions by varying the parameters, the point closest to the centre of the printability area corresponds to the ink composition with 25 wt% of ITO.

Actually, the graph in Figure 3 can be parametrised by a set of *We*^1/2^ isolines based on the formula in Equation (1). These hyperbolic isolines are represented as straight lines using logarithmic axes. The similarity of these lines with the diagonal borders of the ABCD area invokes somewhat the old idea of the importance of a single dimensionless number again, *We* number this case. The line AC corresponds exactly to the *We* value 9 (means *We*^1/2^ = 3). The importance of the *We* criterion was raised by Derby himself again in [33], where a corrected version of his original printability graph is published. However, *We* value 4 is used by Derby according to Duineveld et al. [34] as a minimum Weber number for a drop generation thus delineating the border to overcome the surface tension at the exposed nozzle. This value corresponds to the dotted line A’C’ with *We*^1/2^ = 2 in the graph in Figure 3, predicting a larger printability area. On the other hand, the splashing threshold line BD is not characterized by one value of the Weber number only. Actually, *We*^1/2^ linearly varies from 20 for the point B to 13 for the point D. To generalize this lesson, we believe that the exclusiveness of *We* among all the other dimensionless numbers used for describing the printability of inks it is due to the prime importance of the surface tension among all the discussed ink-jet material characteristics. The absolute condition *sine qua non* of printing is the formation of the ink droplets and there is only one physical variable that is the source of the forces forming the droplet spherical shape, and it is the surface tension. It shall be mentioned that if the fluid is viscoelastic, the elasticity will contribute to the drop formation also. Moreover, the Weber number also contains the tool (*A*) and the process (*ν*) characteristics in contrast to the *Z* number which is based on the material characteristics only. In our specific case, we experienced the good printability when decreasing the *We* value below 47.

### 3.2. Annealing Temperature Optimization

**Figure 4:**
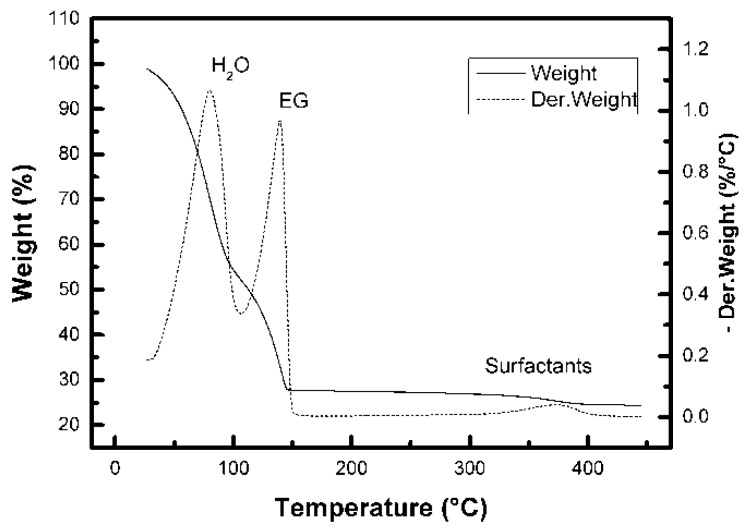
The thermogravimetric curve and its derivative recorded for the ink composition with ITO particle loading 25 wt%.

Optimizing the annealing temperature is the key step in post-printing treatment of the manufactured device. Indeed, this important factor (i) influences grain size, densification or porosity of metal oxide film, which is closely related to the sensing efficiency [8,35,36] furthermore it (ii) determines the resistance of the film and (iii) allows the removal of additives, which could cause interactions with the gas being sensed and thus influence the response of the sensory layer. Finally, the annealing temperature influences (iv) the choice of an appropriate substrate for the final device. Therefore, a thermogravimetric analysis of the prepared ink compositions has been accomplished to observe the temperature ranges corresponding to the evaporation of solvents and the vaporization of surface active polymer additives. A decomposition of the ink proceeded as shown in Figure 4. In the first two steps low-molecular substances (water and ethylene glycol) were volatilized. Their complete evaporation was achieved below a temperature of 200 °C. The complete decomposition step of the present surfactant and the dispersant agents was observed in a range of 300 °C to 400 °C hence the lowest annealing temperature for printed films was set to 400 °C, and other samples were made at 500 °C and 600 °C for comparison too.

**Figure 5:**
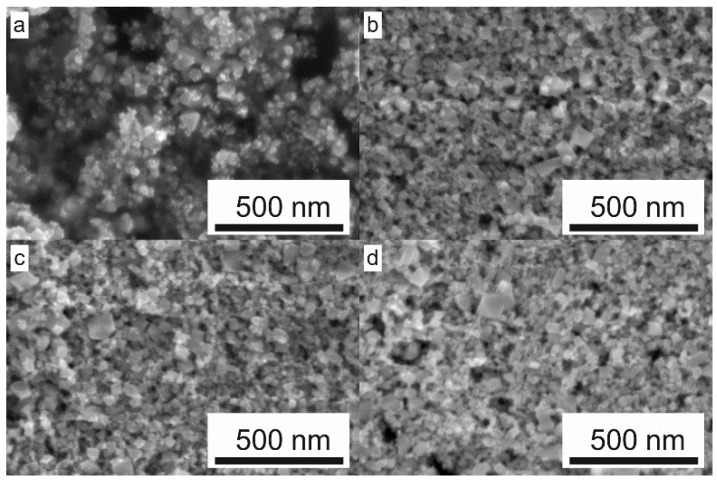
SEM images of ITO particles as received (**a**) and films made from the ink with ITO particle loading 25 wt% annealed to 400 °C (**b**), 500 °C (**c**) and 600 °C (**d**).

**Figure 6:**
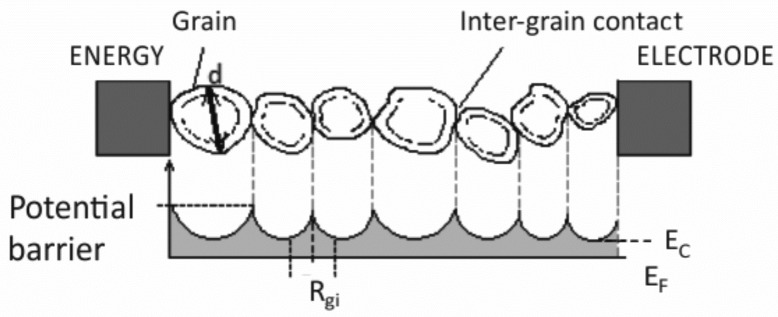
A schematic diagram with energy bands along the sensing layer.

The morphology of original ITO particles as well as annealed films is shown in Figure 5a in the figure depicts the ITO nanopowder as received. Presence of a wide size distribution can be observed. The biggest tetragonal bipyramides are of size below 100 nm but the vast majority of particles is significantly smaller and the powder materials generally corresponds to the specification provided by the supplier (<50 nm particle size). Treating deposited layers at temperatures of 400 °C, 500 °C and 600 °C leads to the formation of separated grains, which are in physical contact, see Figure 5b–d respectively. Formation of short necks between individual grains was not confirmed and could be expected for higher temperatures. Thus, the sensing mechanism can be described using the “grains” or “grain boundary” model, where the Schottky interface between the grains, the height of potential barrier and therefore the dimension of the depletion region variation depends on the ambient atmosphere composition. The conductivity of this treated type of nanocrystalline metal oxide operates by grain boundary space charge (band bending) on inter-grain contact interfaces as shown in Figure 6 where *R_gi_* represents the average intergrain resistance, *d*—the grain diameter, *E_F_*—the Fermi level, *E_C_*—the conductive band [35,37].

**Figure 7:**
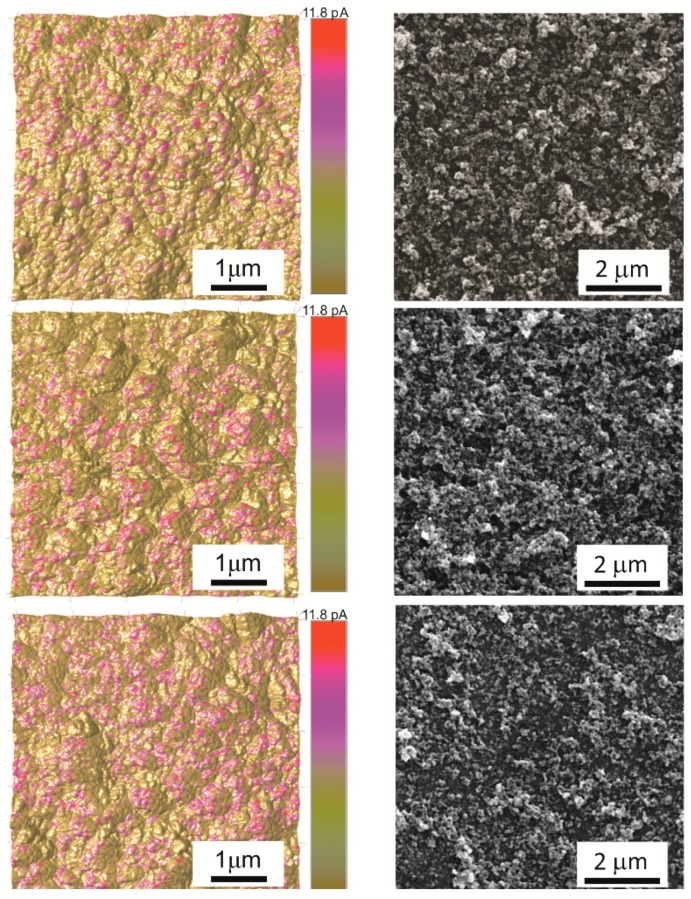
The current mapping (**left side**) and the SEM images (**right side**) on the surface of the printed and annealed films at 400 °C (**top**), 500 °C (**middle**) and 600 °C (**bottom**).

Higher annealing temperatures increase the contact between the grains and can lead to the sintering of the grains in the agglomerates (>500 °C) [38], which contributes to electric transport and increases conductivity. On the other hand, lower annealing temperatures allow for forming a more porous structure and the volume of the layer is more accessible to the detected gas, thus, the active surface is kept [35,37]. As can be seen in Figure 7, the formation of a granular and porous structure is evident. A higher concentration of the charge due to the densification of the structure at a higher annealing temperature of 600 °C, and on the contrary a more porous structure in depth containing less neck connected grains at a lower annealing temperature of 400 °C was observed using scanning tunnelling microscopy combined with surface morphology and scanning electron microscopy of printed and heated films.

### 3.3. Ink-Jet Deposited Films Characteristics

**Figure 8:**
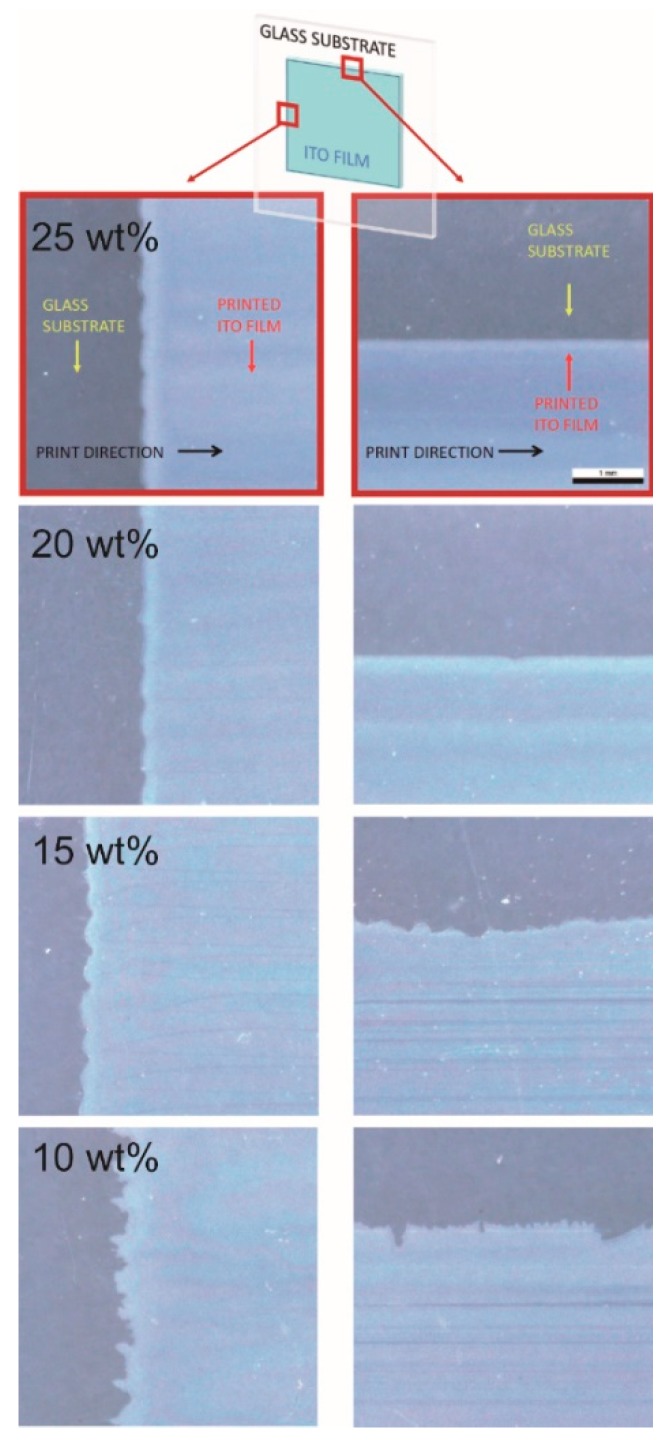
Optical micrographs of one printed layer ITO rectangular motifs. Pairs of magnified edges of squares printed by different ITO concentration inks are presented. The side edge is always in the left image and the top edge is always in the right image. The locations of imaged areas on the specimen, substrate and film material identification, and printing direction are indicated by arrows in the first row of images and this orientation pattern applies for all other rows also.

In general, in comparison to vapour deposition methods, ink-jet material printing does not allow for preparing highly homogenous and flat surfaces (depending on the nature and composition of the ink), which may not be a disadvantage for sensing films, due to the formation of a porous structure. For sensing devices fabricated by an ink-jet printing method it is necessary to achieve high-quality and compact layers which are conductive throughout their volume. A set of representative optical micrographs of printed films is shown in Figure 8. Magnified images of edges of printed rectangular motifs were used to overall quality assessment of the printing. Best contours of the motif were obtained with the 25 wt% ITO ink, hence it performed best among tested dispersions. Therefore, this ink was chosen for printing test specimens and further research. Moreover, Figure 8 documents the high compactness of the printed layer achieved by optimal selection of the ink composition, tuning of the printing process and appropriately selected drop-spacing. An increased amount of nanoparticles in the ink composition increases the thickness of the deposited layers linearly. This finding could lead to the setting of the required thickness through the concentration or loading of particles in the ink. From the obtained values of surface roughness, which decreases with the amount of particles in the ink, it can be concluded that higher loading and a constant annealing temperature leads to the densification of the deposited layer due to easier access and the formation of the grains during the annealing process, which is confirmed by the decrease in bulk resistivity. The densification of the layer and therefore a reduction in porosity may not always be desirable for sensor layers. The data in Table 3 shows that the resistivity of the prepared ITO layers decreases with increasing density of the ink due to an increased concentration of ITO nanoparticles.

### 3.4. Gas Sensing Test

Based on the semiconductor properties of indium tin oxide, sensory behaviour is categorized as electrical and electrochemical receptor or/and transducer. Indium tin oxide, as a degenerate type semiconductor, due to present oxygen vacancies and by Sn doping [7], reacts depending on ambient atmosphere composition by changing the electrical conductivity. The electrical conductivity is transferred as a signal of the electrical resistance variation of the deposited film upon the introduction of reducing or oxidizing gases. The signal of electrical resistance increases/decreases depending on the metal oxide nature as a function of the partial pressure of target gas. The measured electrical resistance is calculated as a sensor response ratio *S_R_* expressed as a percentage as defined [21]:

(2) $$S_{R}\left(\%\right)=\left(\frac{R_{gas}}{R_{air}}-1\right)\cdot 100\%$$

where *R_air_* represents the sensor resistance in air free of vapours atmosphere conditions and *R_gas_* resistance upon vapours ambience.

The mechanism responsible for the changes in the conductivity/resistivity of the layer observed in the presence of vapours can be attributed to predominating models: oxygen ionosorption and oxygen-vacancy model (reduction-reoxidation mechanism). The ionosorption mechanism of the metal oxide sensor response to the gases assumes a reaction of exposure gases with directly pre adsorbed O_2_ species, from ambient oxygen, on the film surface. Oxygen molecules capture free excess electrons from the valence band of the oxide lattice structure and changed as O_2_^−^. This process continues by formation of O^−^ and leads to decreasing the carrier concentration and causes a slight increase in the resistance of the thin film. The reaction is described as follows:

O_2 (ambience)_ + e^−^ → O_2_^−^_(surface)_, (3)

O_2_^−^_(surface)_ + e^−^ → 2O^−^_(surface)_. (4)

The exposed reducing gas reacts with adsorbed oxygen ions on the surface of the sensitive film presented as [3,19,21]:

R + O^−^_(surface)_ → RO + e^−^. (5)

This interaction replenishes the concentration of electrons. Hence, the response of the sensing layer to the reducing gas exposure is manifested as a decrease of its resistance for n-type semiconductors. 

In the case of the oxygen-vacancy model intrinsic defects such as oxygen vacancies on the surface of the metal oxide semiconductor act as electron donors and the surface conductivity is therefore controlled by a reduction and reoxidation of gaseous oxygen. The target gas removes oxygen from the surface side of the lattice and produces oxygen vacancies, which introduce electrons into the conduction band and therefore increases the electrical conductivity. In the absence of a target gas, the oxygen vacancies are filled by gaseous oxygen, and captures electrons resulting in a decrease in the electrical conductivity [3].

**Figure 9:**
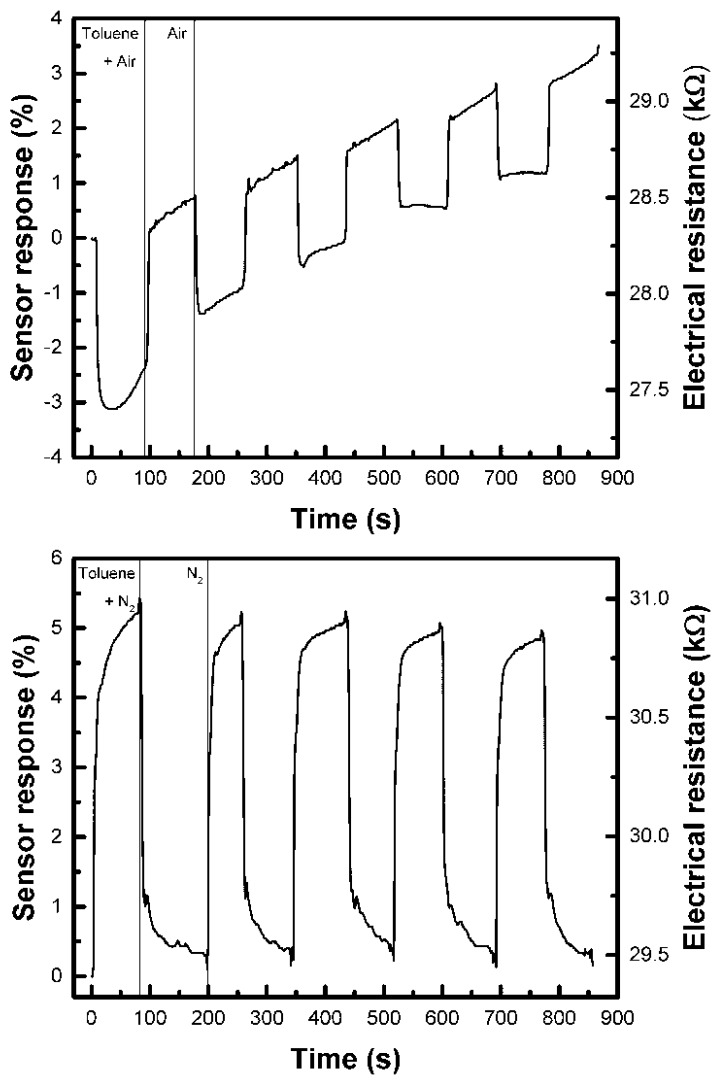
The sensor’s response magnitude to the exposure of Toluene/Air and the saturated vapours of Toluene in an N_2_ ambient atmosphere at 25 °C. The film was prepared from ITO 25 wt% loading ink composition.

In accordance with the above-described mechanisms, we noticed a variation in the electrical resistance of the printed sensors (with a sensing layer prepared from ITO 25 wt% loading ink) upon exposure to saturated vapours of toluene in ambient air (toluene/air) at 25 °C as shown in Figure 9 (upper graph window), which corresponds with the n-type semiconductor behaviour as described by Vaishnav [19]. The response of the sensor operated in air can be explained by both the above-discussed mechanisms. However, the equilibrium in the reactions (3) and namely (4) is shifted to the left side at low temperatures (25–150 °C). The species O^−^ is believed to be dominant at an operating temperature of 300–450 °C while molecular forms should be favourable at low temperatures [39,40]. Therefore, we consider the oxygen vacancy model more plausible for operating the ITO sensor in air atmospheres. Baseline drift can be attributed to the presence of coexisting gases in an uncontrolled ambient laboratory atmosphere since we experienced it regardless to the pre-equlibration of the sensor. Because of the porous nature of the layer as well as a low operating temperature, the sensor requires a longer recovery time needed for the desorption and diffusion of vapour molecules from the porous film. We also observed that resistance increases when the sensor is exposed to saturated toluene vapours in an oxygen-free atmosphere as shown in Figure 9 (lower graph window). This conductivity behaviour inversion can be attributed to a change to the sensing mechanism after placing the sensor into an N_2_ atmosphere similarly as in [3,41,42,43]. However, none of the above-discussed models can apply for this case. It can be reasonably expected that oxygen adsorbates likely desorb from the surface in inert atmosphere, leaving behind electrons to the bulk of the semiconductor. On the other hand, the adsorption of electron accepting gas molecules causes a charge carrier transfer resulting in band bending and the formation of an electron-depleted region (so called space-charge layer), which is manifested as an increase in resistance. It is noteworthy that in a controlled inert atmosphere no baseline drift is experienced. The overall sensitivity of the prepared devices is in order of several % in both types of atmospheres (slightly higher in N_2_) as measured at room temperature which can be explained by a high intrinsic conductivity of the ITO material and a suboptimal thickness of the sensing layer. The charge carrier density of ITO is reasonably high and the shape of the conduction band at the Fermi level faithfully retains the intrinsic parabolic character due to the contribution of Sn 5s electrons. This unique material property makes ITO a highly degenerate n-type semiconductor or, alternatively, a low-carrier-concentration metal [44]. The Debye length of ITO is very small and therefore also the depletion layer on the particle surface is extremely thin hence the main contribution to the sensing layer conductivity is due to the grain volumes which is not affected by the stimulating gas adsorption/desorption [40]. On the other hand, a fast response and recovery of the sensor resistance was experienced, and the performance (sensitivity) of the prepared device can be assessed as very promising if room temperature is considered as the operating temperature.

## 4. Conclusions

There are many adjustable parameters that can significantly affect the ability of the sensing films prepared by using ink-jet printing technology. Besides the tuning of the ink, optimizing the deposition conditions for printing process and compatibility with the substrate used for the sensor devices, therefore several parameters remain crucial: the concentration of the functional material in the ink, which determines the thickness of the final layer, and subsequently, an adequate annealing temperature which can control the porosity of the final film structure.

We demonstrated a successful development of a stabilized indium tin oxide nanoparticles based ink in an aqueous medium suitable for ink-jet printing technology. Dimensionless correlations were used for the analysis of the material-tool-process parameter space. Among the single criterion approaches, the importance of the Weber number was re-discussed and raised again. On the other hand, the description of the processing window requires at least a two dimensional map. We refrained from incorporating the viscoelasticity into our models in favour of reemploying older yet still valuable models limited for Newtonian fluids only. The presented approach enables fast advances in ink formulations and process development with the used DOD printer and a simple experimental instrumentation.

The prepared ink is applicable for the deposition of conductive and transparent thin (about 500 nm) or thick (microns) films depending on the nanoparticles loading. Only one printing run assured a sufficient final quality of films. On the ink-jet printed ITO sensor demonstration specimens, we observed fast response and recovery times to toluene vapours at laboratory temperature although with relatively small overall sensitivity. The type of ITO sensor response depends on the kind of atmosphere under which the sensor is operated in. In air atmospheres, even the low temperature sensing mechanism can be explained by well-established models for n-type semiconductors, among them the oxygen vacancy model seems to be the most favourable. To explain the sensing response inversion observed for the sensor operated in an oxygen-free atmosphere, the charge transfer mechanism between the adsorbent and adsorbate gas molecules was proposed. The overall sensitivity of the device was slightly larger and the sensor response was more stable over the tested number of cycles in pure nitrogen than in an open air ambient. No baseline drift was experienced in a protective atmosphere unlike in air.

The ink-jet printing technique was confirmed as a very promising method for fabricating sensing devices for the gas sensor industry due to its simplicity, rapid and easy prototyping and low cost due to such a versatile material as ITO is. Nevertheless, the preparation of sensors with stable response and enhanced sensitivity to toluene at room temperature remains still a challenging issue.

## Figures and Tables

**Table 1:** Main properties of ITO inks.

Nanoparticles Loading/wt%	10	15	20	25
Surface tension/mN∙m^−1^	21.6 ± 0.1	21.6 ± 0.1	21.6 ± 0.1	21.7 ± 0.1
Density/kg∙m^−3^	1127.6	1186.7	1241.9	1306.6
Viscosity/mPa∙s	3.078	3.518	4.128	4.703

**Table 2:** Calculated dimensionless criteria of prepared inks.

Nanoparticles Loading/wt%	10	15	20	25
*Re*	47.26	43.51	38.81	35.84
*We*	40.44	42.50	44.42	46.60
*Oh*	0.13	0.15	0.17	0.19
*Z*	7.43	6.67	5.82	5.25
*Ca*	0.86	0.98	1.14	1.30

**Table 3:** Thickness, resistivity and surface roughness of one-run printed layers of different ITO inks loading after an annealing process at 400 °C.

Nanoparticles Loading/wt%	10	15	20	25
Film thickness/nm	550 ± 30	780 ± 30	1140 ± 30	1510 ± 40
Resistivity/Ω∙cm	51.88 ± 0.02	45.76 ± 0.03	40.37 ± 0.02	14.21 ± 0.01
Roughness_RMS_/nm	41 ± 3	31 ± 1	31 ± 2	34 ± 1

## Appendix A. Used Equations for Dimensionless Criteria

(A1) $$Re=\frac{\nu \cdot \rho \cdot A}{\eta }$$

where ρ is the density of the fluid, ν is its velocity, η is its dynamic viscosity and *A* is a characteristic length (the diameter of the nozzle).

(A2) $$We=\frac{\nu^{2}\cdot \rho \cdot A}{\sigma }$$

where σ is the surface tension of the fluid.

(A3) $$Oh=\frac{\sqrt{We}}{Re}=\frac{\eta }{\sqrt{\sigma \cdot \rho \cdot A}}=Z^{-1}$$

(A4) $$Ca=\frac{We}{Re}=\frac{\nu \cdot \eta }{\sigma }$$
